# Bacterial communities in domestic washing machines associated with user-perceived odours

**DOI:** 10.1007/s00253-026-13972-1

**Published:** 2026-07-28

**Authors:** Sabasadat Seyednikkhoo, Mirko Weide, Markus Egert

**Affiliations:** 1 Faculty of Health, Medical and Life Sciences, Institute of Precision Medicine, Microbiology and Hygiene Group, Furtwangen University, Villingen-Schwenningen, Germany; 2 Microbiology, International R&D, Henkel Consumer Brands, Henkel AG & Co. KGaA, Düsseldorf, Germany

**Keywords:** Microbial community profiling, Microbial diversity, Nanopore sequencing, Hygiene, Household

## Abstract

Washing machines are known to contain significant amounts of microorganisms with negative implications for laundry and machine hygiene. In particular, the formation of machine and laundry odour and the potential role that microorganisms might play are still far from being understood. Here, we used Oxford Nanopore–based sequencing of full-length 16S rRNA gene amplicons and quantitative PCR to qualitatively and quantitatively investigate the bacterial community composition of different parts (detergent drawer, door seal, sump) of machines categorized as odorous (*n* = 8) and non-odorous (*n* = 8) by their users. DNA was extracted from swab samples taken in a standardized manner from each machine. In total, full-length 16S rRNA gene sequences (~ 1.5 kb) were obtained from the investigated machines and assigned to 1492 distinct species. The observed community compositions corroborated previous investigations using shorter gene sequences for identification. For instance, odour-related *Moraxella osloensis* showed the highest relative abundance in door seal samples. However, only a few statistically significant differences were found in overall community composition (alpha- and beta-diversity) between odorous and non-odorous machines. To investigate whether odorous machines might contain a higher microbial load, bacterial DNA was quantified by qPCR. Odorous machines showed higher median bacterial DNA concentrations across compartments, but odour category had no significant effect on bacterial abundance in a mixed-effects analysis. In summary, odorous and non-odorous machines did not differ greatly in bacterial community composition. Differences in bacterial activity or subjective odour perception may be responsible for perceived odour. Hence, future studies should include metatranscriptomic analyses and larger sample sizes.

## Highlights

• *Odorous washing machines were analyzed with full-length 16S sequencing and qPCR.*

• *Odorous and non-odorous machines differed only slightly in community composition.*

• *Detergent drawer communities showed the strongest association with odour status.*

## Introduction

The conditions in washing machines, including high humidity and nutrient-rich residues, create an ideal environment for microbial growth (Sun et al. 2024). As a result, distinct microbial communities develop under the varying conditions present in different parts of the machine (Nix et al. 2015). These microorganisms can form biofilms, particularly on materials such as polypropylene and rubber, which may contribute to odour development (Munk et al. 2001; Gattlen et al. 2010). Worn clothing, rinse water, and air continuously introduce microorganisms into washing machines, sustaining resilient microbial communities and their associated biofilms. These biofilms are linked to odours and the spread of potential pathogens (Callewaert et al. 2015). Despite this growing concern, the microbial ecology of washing machines and laundered textiles remains poorly understood. Knowledge regarding both the structure (species composition) and function (genetic potential, metabolic activity) of these microbiotas is limited, and the mechanisms by which microbial activity causes laundry odour remain largely unresolved (van Herreweghen et al. 2020). Culture-based methods remain a standard approach for investigating microorganisms in washing machines (Cao et al. 2024). For example, Jacksch et al. (2021) reported an average of (2.1 ± 1.0) × 10^4^ colony-forming units (CFU) cm⁻^2^ across samples from different sites such as the detergent drawers and rubber door seals. Using culture-based approaches, various bacteria have been identified, including *Staphylococcus* spp.*, Bacillus cereus*, and *Kocuria rhizophila* (Jacksch et al. 2021; Li et al. 2016). However, many bacteria exist in a viable but non-culturable (VBNC) state, and plate culture methods capture only a subset of the microbial diversity present in washing machines (Cao et al. 2024). In recent years, amplicon-sequencing technologies have been increasingly applied, as they can detect VBNC bacteria and provide a more comprehensive view of microbial community composition (Cao et al. 2024). Studies by Sun et al. (2024) and Nix et al. (2015) reported that the dominant bacterial phyla — *Pseudomonadota*, *Actinomycetota*, *Bacillota*, and *Bacteroidota* — were present across machine components, including door seals and detergent drawers. In a molecular study based on 16S rRNA gene pyrosequencing of 13 domestic washing machines, Jacksch et al. (2019) identified the same dominant phyla and reported the detergent drawer as the site with the highest bacterial diversity and the door seal as the site with the highest relative abundance of odour-forming *Moraxella osloensis*. Despite these findings, the relationship between microbial composition and machine odour remains unclear. In the present study, we addressed this gap by combining Oxford Nanopore–based full-length 16S rRNA gene sequencing with quantitative PCR to investigate the qualitative and quantitative bacterial community composition in different machine compartments (detergent drawer, door seal, and sump). Machines were categorized as odorous or non-odorous by their users. This approach enabled high-resolution species identification and comparison of bacterial loads between machines with and without odour, providing a more comprehensive understanding of potential microbial contributors to laundry and machine hygiene issues.

## Material and methods

### Selection and sampling of washing machine

A total of 16 in-use domestic washing machines were examined in this study, comprising eight with user-reported odour (“odorous”) and eight without odour (“non-odorous”). Recruitment was based on a university-wide call for participation. Although additional households expressed interest, only 16 washing machines fulfilled the study criteria and could be included in the final dataset. Swab samples were collected from coworkers and students at Furtwangen University who resided within a 50-km radius of Villingen-Schwenningen, including the cities of Furtwangen and Tuttlingen, Germany. Sampling was conducted at three selected sites of machines: detergent drawer, rubber door seal, and the sump. Sampling was performed primarily by the first author, with occasional assistance from a master’s student under direct supervision to ensure consistency in technique. For the detergent drawer, samples were taken along the edges of each filling compartment, starting from the outer right edge of the right container and moving along the perimeter to the left edge, including corners where residues may accumulate; the same procedure was applied for the middle and left compartments when present. For the rubber door seal, sampling started at the centre of the porthole and proceeded along the seal to the lowest point, swabbing approximately 15 cm to the right and left from the starting point while also collecting material from under the seal. For the sump, the chamber was opened, and samples were collected from the walls of the inner part of the sump to capture residues adhering to the surface. For each sampling site, one swab was collected for full-length 16S rRNA gene amplicon sequencing and a second independent swab was collected for quantitative PCR (qPCR). Sampling areas were standardized to enable quantitative comparison: 5 cm^2^ for the detergent drawer, 1 cm^2^ for the door seal, and 1 cm^2^ for the sump. Sterile swabs (Medical Wire & Equipment, Corsham, UK) stored in RNA/DNA Shield buffer (Zymo Research, Freiburg, Germany) were used to ensure nucleic acid stabilization. Samples were stored at − 20 °C prior to further processing.

### DNA extraction

Field blanks consisted of sterile swabs opened at the sampling site but not used for surface sampling. These blanks were transferred to RNA/DNA shield and processed together with all samples throughout the workflow. DNA concentrations in all blanks were very low and below the detection limit of Qubit (< 0.050 ng/ml). DNA was extracted from swabs using the ZymoBIOMICS DNA Miniprep Kit (Zymo Research) with bead-beating in a FastPrep instrument (MP Biomedicals, Eschwege, Germany), following the manufacturer’s protocol. Briefly, swabs were placed in RNA/DNA Shield (Zymo Research) for nucleic acid stabilization and then transferred directly into ZR BashingBead tubes from the same kit for mechanical lysis. After centrifugation with MiniSpin centrifuge (Eppendorf, Hamburg, Germany), the supernatant was mixed with DNA binding buffer to promote adsorption of nucleic acids to the silica membrane of spin columns. Bound DNA was purified through two sequential washing steps and eluted in 50 µl DNase/RNase-free water. Extracted DNA was subsequently used for full-length 16S rRNA gene amplicon sequencing (Oxford Nanopore platform) and for quantitative PCR (qPCR) analysis.

### PCR amplification of 16S rRNA genes and cleanup

Bacterial community composition was assessed using amplicon sequencing of the 16S rRNA gene. Library preparation was performed with the Rapid PCR Barcoding Kit 24 V14 (SQK-RPB114.24, Oxford Nanopore Technologies, Oxford, UK). The kit is based on a transposase enzyme that simultaneously fragments template DNA molecules and attaches tags containing primer-binding sites to the cleaved ends. It includes 24 primers, each with a unique barcode and a 5′ tag, enabling the ligase-free addition of Rapid Sequencing Adapters (Oxford Nanopore Technologies). PCR amplification was carried out in a total volume of 50 µl, consisting of 15 µl of input DNA, 25 µl of LongAmp Hot Start Taq 2 × Master Mix (New England Biolabs, Frankfurt, Germany), and 10 µl of the corresponding 16S barcode primer. Reactions were performed on a T100 Thermal Cycler (Bio-Rad Laboratories, Singapore, Singapore) under the following conditions: initial denaturation at 95 °C for 1 min; 25 cycles of denaturation at 95 °C for 20 s, annealing at 55 °C for 30 s, and extension at 65 °C for 2 min; and a final extension at 65 °C for 5 min. Amplicon size was confirmed by electrophoresis on 1% agarose gels stained with Midori Green Advance (NIPPON Genetics EUROPE, Germany). Blanks were subsequently subjected to the same PCR amplification and barcoding protocol as the samples; however, post-PCR gel electrophoresis showed no visible bands, and Qubit measurements confirmed that DNA concentrations remained below the detection limit (< 0.050 ng/µl), indicating negligible contamination. PCR products were purified using AMPure XP magnetic beads (Beckman Coulter, USA) according to the manufacturer’s instructions, including two washing steps with 80% ethanol, using a HulaMixer (Thermo Fisher Scientific, Frankfurt, Germany) and MiniSpin centrifuge. DNA was eluted in 15 µl of the kit-provided elution buffer, and centrifugation steps were performed with the MiniSpin centrifuge. The final library was loaded onto a flow cell and sequenced on a GridION device (Oxford Nanopore Technologies).

### qPCR-based quantification of bacterial DNA

For quantitative PCR (qPCR) targeting the 16S rRNA gene, DNA from seven odorous and seven non-odorous washing machines was analysed. DNA from one machine in each group was excluded due to insufficient concentration for reliable quantification. Amplification was performed using the primer pair (Jiang 2018) 8 F (5′-AGTTGATCCTGGCTCAG-3′) and 511R (5′-GTATTACCGCGGCTCCTGCA-3′). The qPCR reaction mixture contained 5 µl of input DNA, 10 µl of PowerUp SYBR Green Master Mix (Thermo Fisher Scientific), 2 µl of DNase/RNase-free water (Zymo Research), 1 µl of each forward and reverse primer (Thermo Fisher Scientific), and 1 µl of Bovine Serum Albumin (BSA, 4 mg/ml; Thermo Fisher Scientific). Primer concentrations were optimized between 300 and 800 nM according to the PowerUp SYBR Green Master Mix protocol for standard mode. The reaction mixtures were loaded into wells of a LightCycler Multiwell Plate 96 (Roche Diagnostics GmbH, Mannheim, Germany), sealed with optical adhesive covers, and centrifuged briefly to remove air bubbles using a Centrifuge 5810 R (Eppendorf GmbH, Hamburg, Germany). Each sample was analysed in triplicate, including a negative control and a seven-point standard series of *Escherichia coli* DNA, ranging from 17.5 to 0.000112 ng/µl with five-fold serial dilutions. qPCR was performed on a LightCycler 480 instrument (Roche Diagnostics Ltd., Rotkreuz, Switzerland) under standard cycling conditions for primers with Tm below 60 °C. The protocol included UDG activation at 50 °C for 2 min, DNA polymerase activation (Dual-Lock) at 95 °C for 2 min, and 40 cycles of amplification with denaturation at 95 °C for 15 s, annealing at 59 °C for 15 s, and extension at 72 °C for 1 min. Melting curve analysis was performed with 95 °C for 15 s at a ramp rate of 1.6 °C/s, 60 °C for 1 min at 1.6 °C/s, and 95 °C for 15 s at 0.11 °C/s. Sampling was conducted separately for the detergent drawer, door seal, and sump of each washing machine, resulting in three 96-well plates per experiment. Absolute quantification was calculated using LightCycler 480 software (version 1.5.0.39) and reported as DNA concentration in ng/µl.

### Bioinformatic and statistical analyses

Sequencing experiments were analyzed using the MinKNOW software (Oxford Nanopore Technologies). The generated FASTQ files were subsequently processed in Epi2ME version 5.2.4 (Oxford Nanopore Technologies) with Minimap2 for classification against the NCBI 16S/18S database. The NCBI 16S/18S database was used because it represents the standard reference database implemented in this workflow. Filtering parameters included a minimum read threshold of 10, ≥ 95% identity to reference sequences, ≥ 90% coverage, and an abundance threshold of 1. While the workflow does not explicitly remove chimeric sequences, the combination of quality and abundance filters, together with negative controls that showed no detectable DNA on Qubit (< 0.050 ng/µl) or gel electrophoresis, indicates that contamination or chimeric sequences are unlikely to have influenced the observed high-abundance taxa or the comparative analyses between odorous and non-odorous washing machines. Further data processing and visualization were conducted in R version 4.4.3 (R Core Team 2025) using RStudio version 2025.5.1.513 (Posit Team 2025). To describe microbial community composition, the relative abundance of species at each sampling site was calculated. Alpha diversity was assessed by calculating species richness, Shannon diversity, and Pielou’s evenness. Statistical comparisons between odorous and non-odorous washing machines were performed using the Wilcoxon–Mann–Whitney *U* test for each alpha diversity index and top 10 most abundant taxa. Beta diversity between samples was assessed using principal coordinate analysis (PCoA) based on weighted and unweighted UniFrac distance matrices. Sample metadata were organized in tab-separated files, linking each barcode to its respective sampling location (detergent drawer, door seal, sump). FASTA outputs from multiple Epi2ME 16S workflow instances were merged into a single reference file, and duplicate sequence identifiers were removed to ensure compatibility with downstream analyses. Deduplicated sequences were imported into QIIME 2 (v2023.2) as FeatureData[Sequence], aligned with MAFFT, and phylogenetic trees were constructed using FastTree. The rooted tree was exported in Newick format, and OTU tables and phylogenetic tree tip labels were processed in R to ensure correspondence between taxa and tree tips. Only taxa shared between the OTU table and the phylogenetic tree were retained using phyloseq (v1.42.0). Weighted and unweighted UniFrac distances were then calculated, and resulting PCoA ordinations were visualized with ggplot2 (v3.4.4). We used analysis of similarities (ANOSIM) and permutational multivariate analysis of variance (PERMANOVA) with 9999 permutations to check whether samples show statistically significant differences in community structure at the different sampling sites and smell groups. To account for the non-independence of samples originating from the same washing machine, qPCR data were additionally analysed using a linear mixed-effects model. Odour category (odorous vs. non-odorous) and sampling position (detergent drawer, door seal, sump) were included as fixed effects, whereas washing machine identity was included as a random effect. Because qPCR values showed substantial variability, data were log10-transformed prior to analysis. Models were fitted using the lme4 and lmerTest packages in R. Pairwise comparisons of low-abundance taxa were conducted separately for each sampling position using Wilcoxon rank-sum tests on relative abundance data. Taxa were included only if present in at least two samples within either odour group at the respective sampling position.

## Results

### Bacterial community composition in different parts of odorous vs. non-odorous machines

**Fig. 1:**
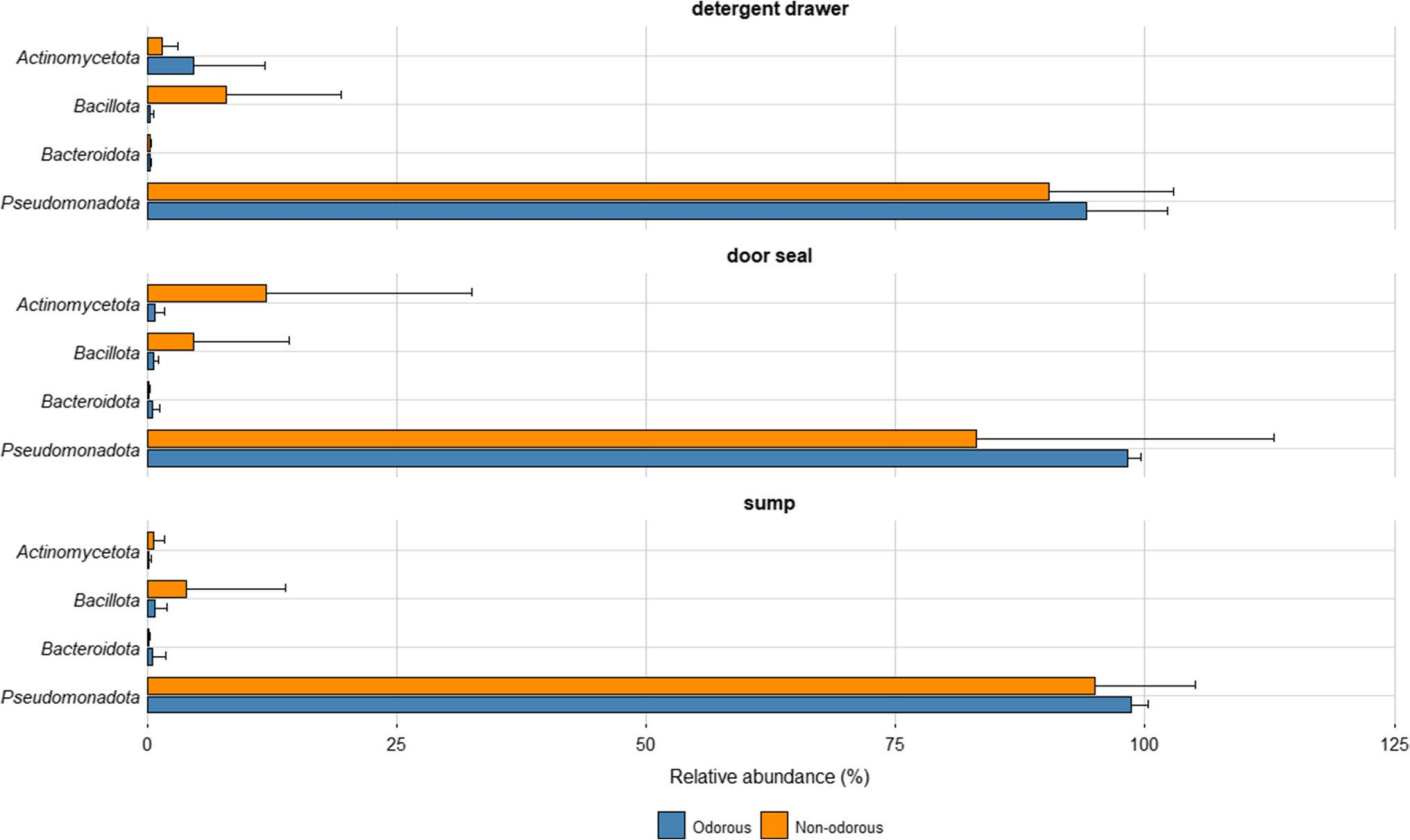
Relatively most abundant bacterial phyla in three different washing machine compartments of odorous (blue) and non-odorous (orange) machines. Bars represent mean relative abundance (%) + SD. Significance was assessed using the Wilcoxon–Mann–Whitney *U* test (*p < 0.05; **p < 0.01; ***p < 0.001). The absence of a symbol indicates no significant difference

At the phylum level (Fig. 1), bacterial community composition differed between odorous and non-odorous washing machines across the three sampling sites. In all locations, *Pseudomonadota* was the dominant phylum in both odorous and non-odorous groups. Odorous washing machines exhibited a higher relative abundance of *Pseudomonadota* compared to non-odorous machines; however, this difference was not statistically significant at any site.

**Fig. 2:**
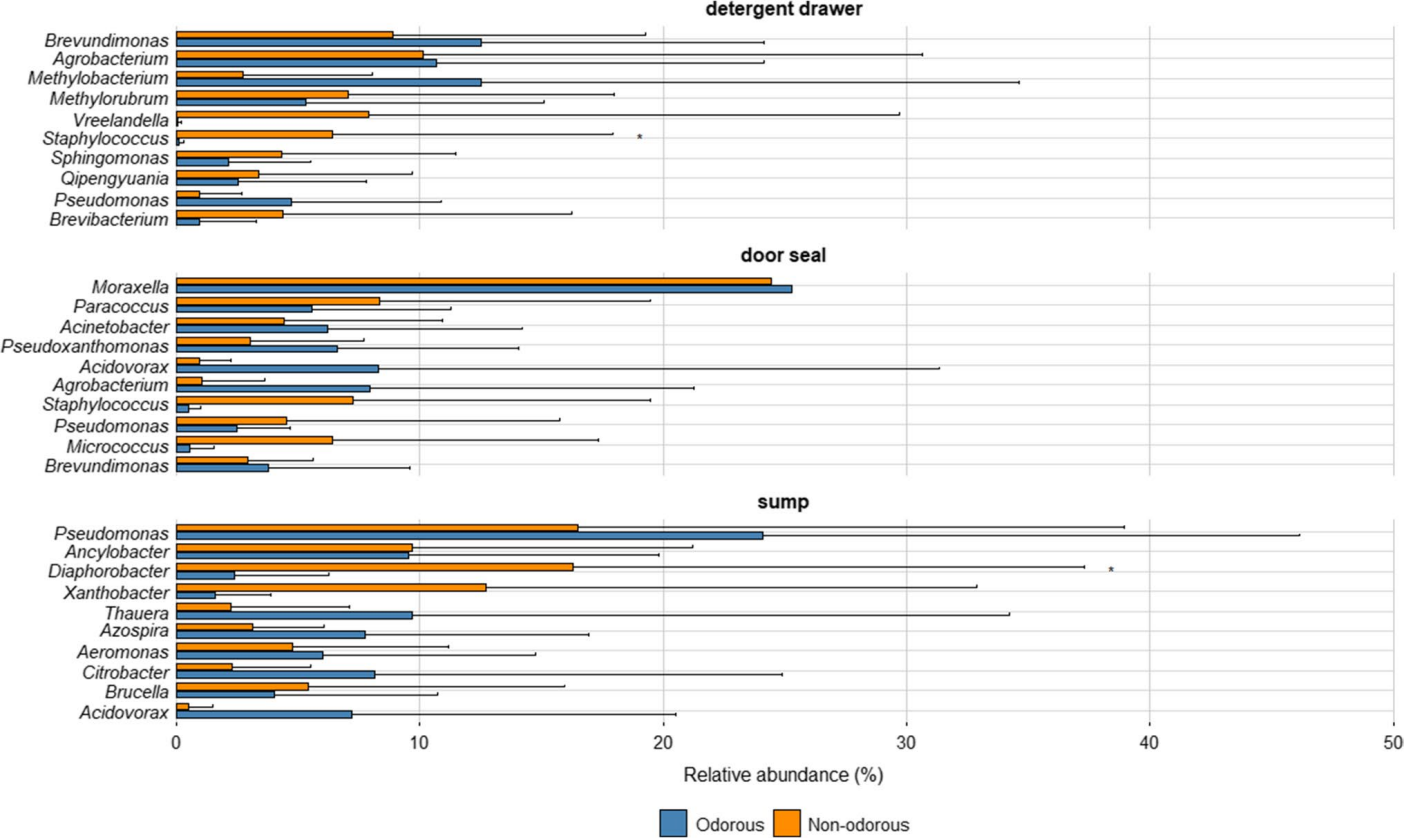
Top 10 most abundant bacterial genera in three different washing machine compartments of odorous (blue) and non-odorous (orange) machines. Bars represent mean relative abundance (%) + SD. Significance was assessed using the Wilcoxon–Mann–Whitney *U* test (*p < 0.05; **p < 0.01; ***p < 0.001). The absence of a symbol indicates no significant difference

At genus level (Fig. 2), microbial communities exhibited distinct compartment-specific patterns, but overall differences between odorous and non-odorous washing machines were limited. In the detergent drawer, the most abundant genera were *Agrobacterium*, *Brevundimonas*, and *Methylobacterium*, followed by *Methylorubrum*, *Vreelandella*, and *Staphylococcus*. While most genera showed no significant differences between odorous and non-odorous machines, *Staphylococcus* was significantly higher in the non-odorous group. In the door seal, the community was relatively dominated by *Moraxella*, which accounted for almost 20% of the total relative abundance across both groups, with additional contributions from *Paracoccus*, *Acinetobacter*, *Pseudoxanthomonas*, and *Acidovorax*. No significant differences were detected between odorous and non-odorous machines for any of the genera in the door seal compartment. In sump, *Pseudomona*s was the most dominant genus, with relative abundances ranging from approximately 16% to 24% across samples, followed by *Ancylobacter*, *Diaphorobacter*, *Xanthobacter*, and *Thauera*. Of these, *Diaphorobacter* was significantly more abundant in non-odorous machines, while all other genera showed no significant differences.

**Fig. 3:**
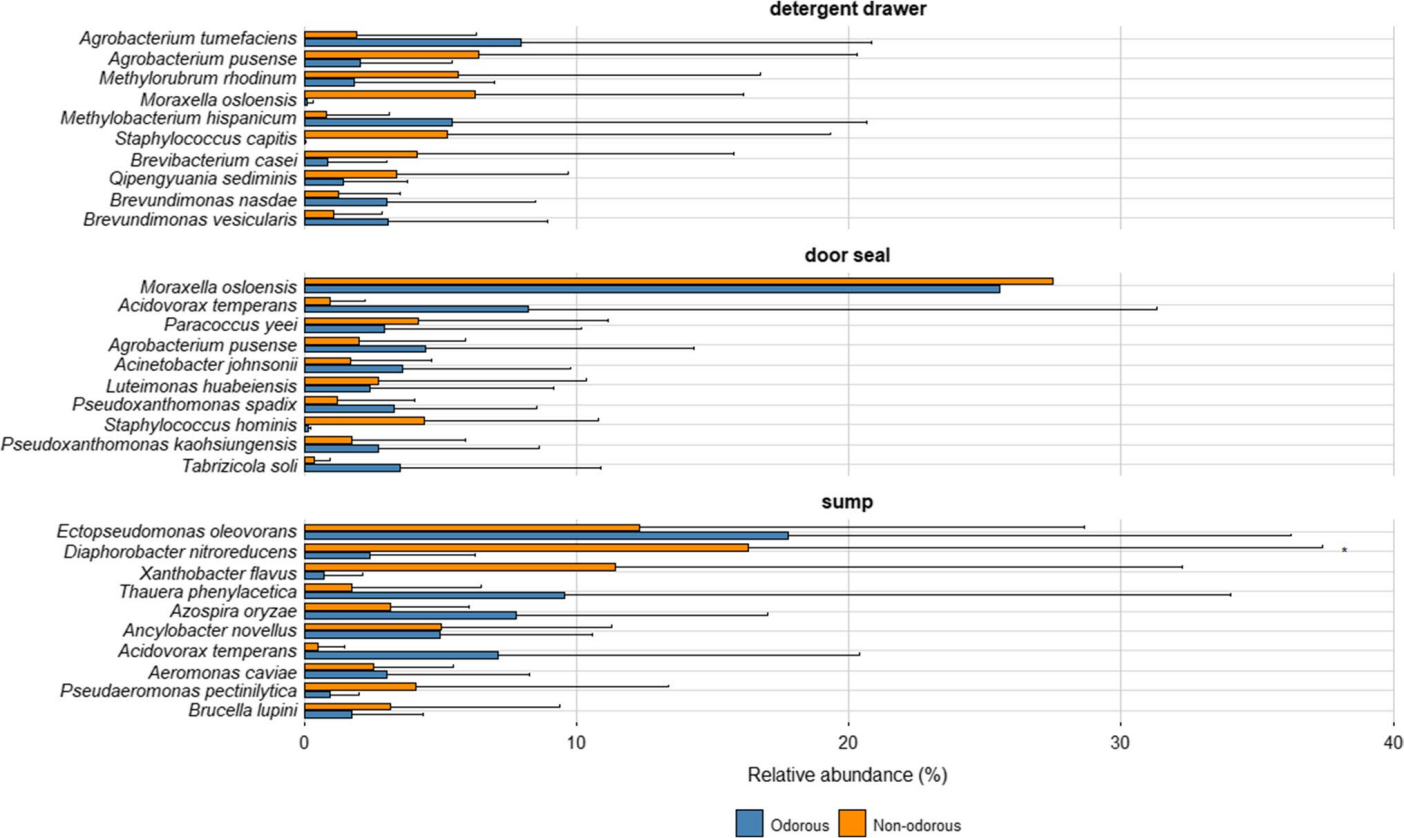
Top 10 most abundant bacterial species in three different washing machine compartments of odorous (blue) and non-odorous (orange) machines. Bars represent mean relative abundance (%) + SD. Significance was assessed using the Wilcoxon–Mann–Whitney *U* test (*p < 0.05; **p < 0.01; ***p < 0.001). The absence of a symbol indicates no significant difference

At the species level (Fig. 3), the most abundant taxa again varied by compartment. In the detergent drawer, communities were composed of multiple genera-level representatives without a single dominant species (Fig. 3). In the door seal, *Moraxella osloensis* relatively dominated both odorous and non-odorous machines, with no significant differences detected. In the sump, *Ectopseudomonas oleovorans* and *Diaphorobacter nitroreducens* were the dominant species. Notably, *Diaphorobacter nitroreducens* was significantly higher in non-odorous sumps.

### Alpha diversity across locations and smell groups

**Fig. 4:**
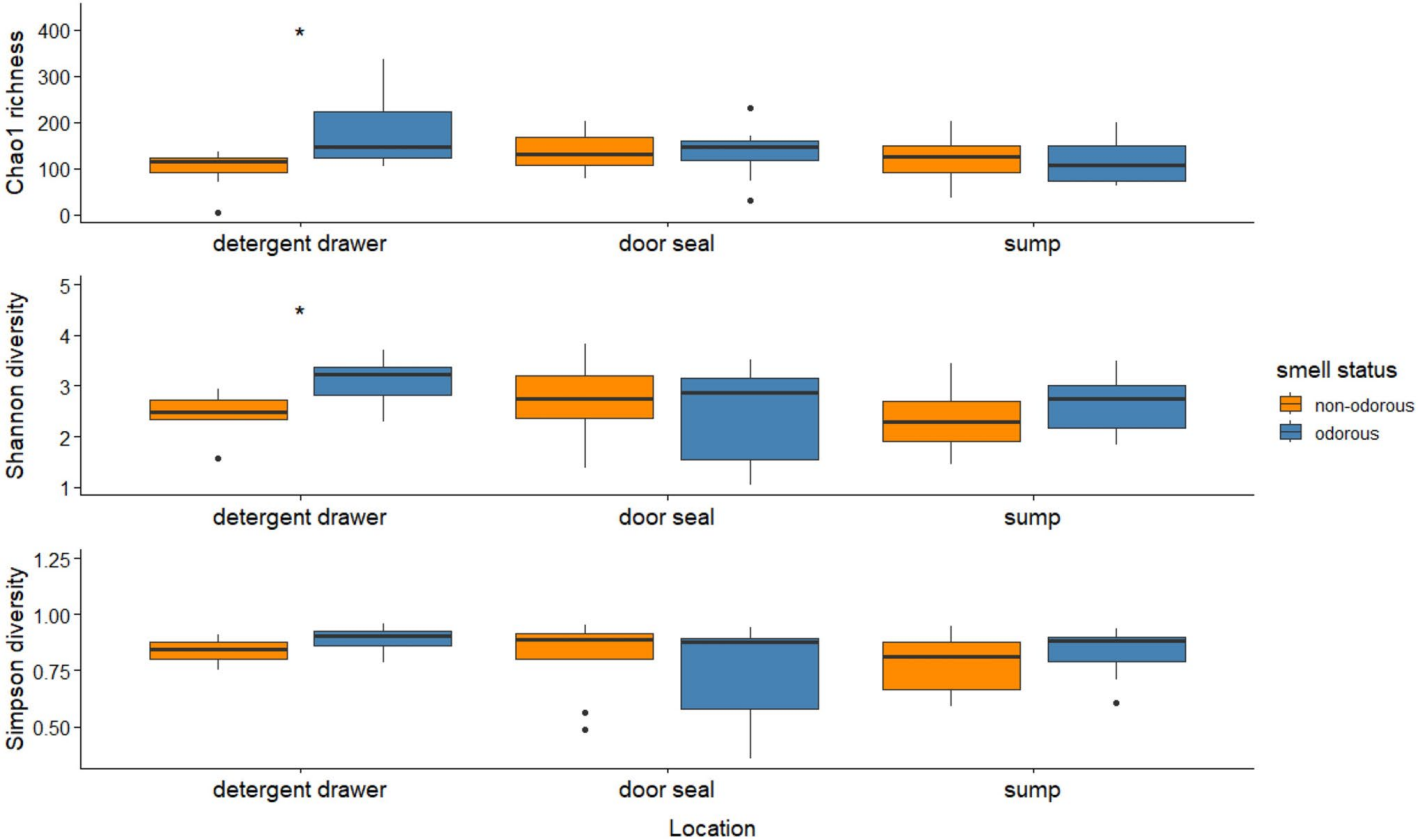
Boxplots for the distribution of alpha diversity measures comparing sampling sites and smell of washing machines. Colours indicates smell group: odorous (blue), non-odorous (orange). Statical analysis was done using the Wilcoxon-Mann–Whitney *U* tests for independent samples. *p*-values are indicated by asterisks (*p < 0.05). The absence of a symbol indicates no significant difference

Alpha diversity analyses based on Chao1 richness, Shannon diversity, and Simpson’s evenness indices were conducted to compare microbial community structures between odorous and non-odorous washing machine samples across three locations (Fig. 4). Alpha diversity patterns varied only slightly among sampling locations. Based on mean values, detergent drawer samples exhibited the highest Chao1 richness (mean = 139), Shannon diversity (mean = 2.78), and Simpson diversity (mean = 0.865), followed by door seal and sump samples. In contrast, median values for all three indices were highest in door seal samples. Comparison between odorous and non-odorous washing machines revealed significantly higher Chao1 richness and Shannon diversity in odorous detergent drawer samples (*p* < 0.05). No significant differences between odorous and non-odorous machines were observed for door seal or sump samples.

### Beta diversity across locations and smell groups

**Fig. 5:**
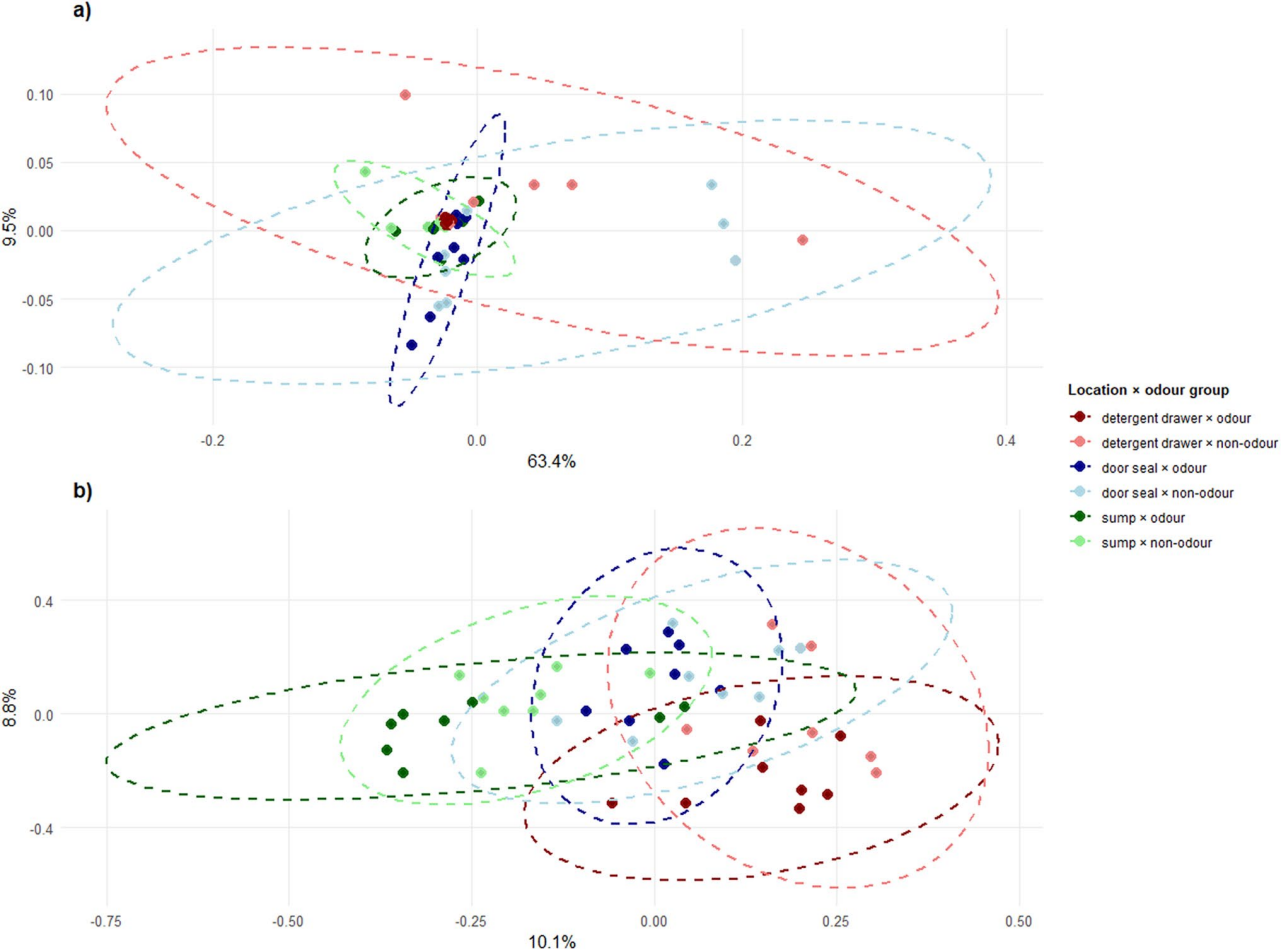
Principal coordinate analysis (PCoA) plot of weighted (**a**) and unweighted (**b**) Unifrac measures using the 16S rRNA gene sequencing data of 48 analysed washing machine samples. Colours indicate sampling site and smell group: door seal (blue range), detergent drawer (red range), sump (green range)

Principal coordinate analysis (PCoA) based on weighted and unweighted UniFrac distances was performed to visualize differences in microbial community structure between odorous and non-odorous washing machine samples across locations. The weighted UniFrac analysis (Fig. 5a) accounts for both phylogenetic relationships and relative abundances of taxa. In contrast, the unweighted UniFrac analysis (Fig. 5b) considers only the presence or absence of taxa. Pairwise analyses comparing odorous and non-odorous samples within each washing machine compartment revealed location-specific differences in bacterial community structure. In the sump, no significant separation was observed between odorous and non-odorous samples for either weighted or unweighted UniFrac distances (weighted UniFrac: ANOSIM *R*^2^ = 0.041, *p* = 0.172; PERMANOVA *R*^2^ = 0.061, *p* = 0.584; unweighted UniFrac: ANOSIM *R*^2^ = −0.003, *p* = 0.471; PERMANOVA *R*^2^ = 0.063, *p* = 0.566), indicating largely similar microbial communities. In the detergent drawer, significant differences were detected between odorous and non-odorous samples in weighted ANOSIM and unweighted PERMANOVA (weighted UniFrac: ANOSIM *R*^2^ = 0.215, *p* = 0.004; PERMANOVA *R*^2^ = 0.170, *p* = 0.055; unweighted UniFrac: ANOSIM *R*^2^ = 0.186, *p* = 0.054; PERMANOVA *R*^2^ = 0.092, *p* = 0.037). The door seal exhibited significant differences in unweighted ANOSIM and PERMANOVA (unweighted UniFrac: ANOSIM *R*^2^ = 0.207, *p* = 0.028; PERMANOVA *R*^2^ = 0.091, *p* = 0.037, weighted UniFrac: ANOSIM *R*^2^ = 0.065, *p* = 0.246; PERMANOVA *R*^2^ = 0.185, *p* = 0.126). Overall, these results indicate that microbial variation between odorous and non-odorous samples is most evident in the detergent drawer and door seal, while the sump shows little difference.

### Species associated with differences in community composition

Because unweighted UniFrac analyses are sensitive to differences in community membership, species-level comparisons were performed to identify taxa differing significantly in relative abundance between odorous and non-odorous washing machines. Species with significantly higher relative abundances in odorous samples are summarized in Table 1.

**Table 1:** Species with significantly higher relative abundances in odorous compared with non-odorous washing machine samples (*p* < 0.05). Species detected in at least two odorous samples within each sampling location were compared using the Wilcoxon–Mann–Whitney test. The reported mean relative abundance (%) represents the average relative abundance of each species across odorous washing machine samples after normalization within individual samples

Location	Species	Mean relative abundance (%)	*p*-value
Detergent drawer	*Pseudorhodoferax soli*	1.92	0.0035
Detergent drawer	*Phenylobacterium haematophilum*	1.34	0.0077
Detergent drawer	*Shinella oryzae*	1.01	0.0127
Detergent drawer	*Stenotrophomonas nematodicola*	0.34	0.0215
Detergent drawer	*Achromobacter mucicolens*	0.27	0.0239
Door seal	*Agrobacterium deltaense*	0.08	0.0127
Door seal	*Pseudomonas sediminis*	0.06	0.0325
Sump	*Ochrobactrum quorumnocens*	0.02	0.0325

### Bacterial load in odorous vs. non-odorous machine based on qPCR

**Fig. 6:**
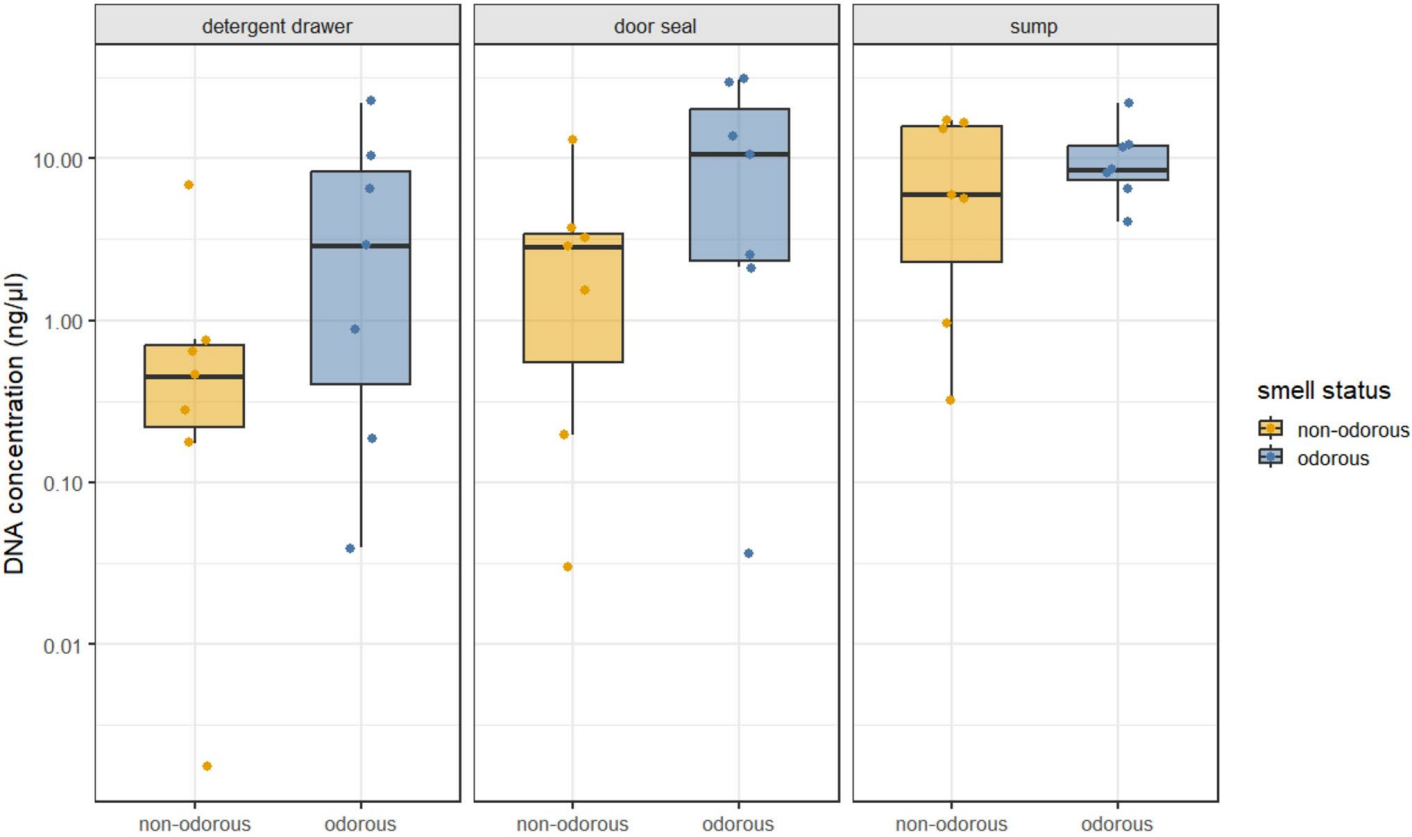
Bacterial DNA concentrations (ng/µl) determined by qPCR in detergent drawer, door seal, and sump samples from odorous (*n* = 7) and non-odorous (*n* = 7) washing machines. Each point represents an individual washing machine sample. Boxes indicate the interquartile range (IQR), the horizontal line within each box represents the median, and whiskers extend to 1.5 × IQR. The *y*-axis is displayed on a logarithmic scale

All samples analysed by qPCR produced amplification curves within the expected range, consistent with the *E. coli* serial dilution standards. Each sample was analysed in triplicate reactions, and the mean values were calculated and further averaged by sampling location (detergent drawer, door seal, and sump) across seven odorous and seven non-odorous washing machines. Reported concentrations were normalized to an equivalent sampled surface area of 1 cm^2^ per location. The qPCR assays demonstrated efficiencies above 90%, with standard curve slopes ranging between −3.4 and −3.5. Individual qPCR measurements are shown in Fig. 6. Although odorous washing machines generally exhibited higher median bacterial DNA concentrations across all three compartments, substantial variability and overlap between odorous and non-odorous machines were observed. A linear mixed-effects model revealed a significant effect of sampling position (*F* = 4.81, *p* = 0.017), whereas odour category was not statistically significant (*F* = 4.04, *p* = 0.067).

## Discussion

One of the main objectives of this study was to qualitatively and quantitatively compare bacterial communities between “odorous” and “non-odorous” household washing machines and to assess whether specific taxa or bacterial loads are associated with perceived odour. To achieve this, we examined 16 different washing machines, sampling three distinct locations within each machine. Bacterial community composition was assessed using full-length 16S rRNA nanopore sequencing, while total bacterial loads were quantified by qPCR.

Using multiple alpha-diversity metrics, we found that detergent drawer samples exhibited the highest bacterial diversity, as reflected by Chao1 richness, Shannon diversity, and Simpson diversity. This observation is consistent with previous studies by Nix et al. (2015) and Jacksch et al. (2019), which also identified the detergent drawer as the washing machine compartment with the highest bacterial diversity. Although median values were slightly higher in door seal samples, mean alpha-diversity values were consistently highest in detergent drawer samples. When comparing odorous and non-odorous washing machines, alpha-diversity metrics revealed only minor differences between smell groups. Chao1 richness and Shannon diversity were significantly higher in odorous detergent drawer samples, whereas no significant differences were observed in door seal or sump samples. These findings suggest that bacterial diversity is primarily determined by sampling location, while odour status exerts only limited and compartment-specific effects. The beta-diversity analyses further support these patterns. Principal coordinate analysis (PCoA) based on weighted and unweighted UniFrac distances indicated similar result. In the sump no significant differences were observed, indicating largely similar microbial community structures irrespective of odour classification. In the detergent drawer, differences between odorous and non-odorous samples were detected across multiple analysis. Weighted Unifrac ANOSIM identified a significant difference between smell group, while the corresponding weighted UniFrac PERMANOVA result was not statically. In the unweighted analysis, PERMANOVA detected significant separation between smell group, whereas ANOSIM showed a near-significant trend (weighted UniFrac: ANOSIM *R*^2^ = 0.215, *p* = 0.004; PERMANOVA *R*^2^ = 0.170, *p* = 0.055; unweighted UniFrac: ANOSIM *R*^2^ = 0.186, *p* = 0.054; PERMANOVA *R*^2^ = 0.092, *p* = 0.037. Collectively, these results indicate that detergent drawer samples from odorous and non-odorous washing machines differ in both weighted and unweighted UniFrac-based community comparisons, depending on the statistical test applied. The door seal exhibited a contrasting pattern, with significant differences between odorous and non-odorous samples detected only in unweighted Unifrac analysis, while weighted Unifrac analyses were not significant (unweighted UniFrac: ANOSIM *R*^2^ = 0.207, *p* = 0.028; PERMANOVA *R*^2^ = 0.091, *p* = 0.037, weighted UniFrac: ANOSIM *R*^2^ = 0.065, *p* = 0.246; PERMANOVA *R*^2^ = 0.185, *p* = 0.126). This suggests that differences associated with odour classification in the door seal are primarily related to variation in community membership rather than shifts in the relative abundance of dominant taxa. To further explore the taxa potentially contributing to the differences observed in the unweighted UniFrac analyses, species-level comparisons identified several taxa with significantly higher relative abundances in odorous washing machines. Among these, *Achromobacter mucicolens* has been reported to possess biofilm-forming capacity, while members of the genus *Pseudomonas* are also well known for their ability to form biofilms and for their role in odour formation. (Al-Kahachi et al. 2023; Carroll et al. 2005). However further studies will be needed to confirm and functionally clarify any potential role of these minor-abundant bacteria for odour formation.

Previous studies have also shown that bacterial communities in washing machines differ depending on the location, as each component has distinct environmental conditions (Sun et al. 2024; Jacksch et al. 2021). In particular, the detergent drawer and fabric softener reservoir create a unique microenvironment where bacteria can utilize detergent components such as fatty acids, surfactants, and alcohols as nutrients, while bleach-containing detergents inhibit growth, promoting biofilm formation and odour persistence (Babič et al. 2015; Whitehead et al. 2025). The phylum *Pseudomonadota* was the relatively most abundant across all sampled sites of the washing machines, with a relative abundance exceeding 75%. This phylum is also predominant in drinking water systems, which likely explains its widespread occurrence throughout the washing machine, as tap water supplies all components (Becerra-Castro et al. 2016; Kwon et al. 2011; Vaz-Moreira et al. 2017). Members of the genus *Pseudomonas*, which are typical inhabitants of drinking water, were particularly dominant in the sump and ranked among the top ten genera in both the detergent drawer and door seal samples (Kwon et al. 2011; McLellan et al. 2015). Genera such as *Moraxella*, which was the most dominant in door seal and *Acinetobacter*, which ranked third in abundance of the same location, are commonly associated with the human skin microbiome and are likely introduced into the washing machine via contact with worn textiles (Al-Khoja and Darrell 1979; Ring et al. 2017). *Moraxella osloensis* was the most abundant species detected on the door seal. This bacterium has previously been associated with unpleasant laundry odours (Munk et al. 2001; Stapleton et al. 2013; Takeuchi et al. 2013). Its tolerance to drying conditions and metabolic capacity to generate volatile compounds such as 4-methyl-3-hexenoic acid are thought to play major roles in both persistence and odour production within washing machines (Kubota et al. 2012). Regular use of detergents formulated with bleaching or disinfecting agents can help reduce microbial growth and limit odour development (Takeuchi et al. 2013). Despite clear compartment-specific differences in bacterial community structure, our analyses revealed no statistically significant differences in the relative abundances of the top 10 most abundant taxa between odorous and non-odorous washing machines at most taxonomic levels. Overall, bacterial community composition appeared largely similar between smell groups, suggesting that odour formation may not be directly linked to the presence or relative abundance of dominant bacterial taxa alone. Notably, taxonomic comparisons in the present study were based on relative abundance data. Although microbiome sequencing datasets are compositional in nature (Gloor et al. 2017), recent evidence suggests that methods specifically developed for compositional data are not universally superior to analyses based on relative abundances (Pelto et al. 2025). Therefore, relative abundance-based analyses were considered appropriate for the exploratory comparisons performed in this study.

Odorous washing machines showed higher median bacterial DNA concentrations across all three compartments. Although bacterial DNA concentrations varied substantially among individual machines and the effect of odour category did not reach statistical significance in the mixed-effects model, sampling position was identified as a significant determinant of bacterial DNA concentrations. Future studies with larger sample sizes will be required to further evaluate the observed differences between odour groups.

Together, our results suggest that machine odour is associated with subtle shifts in microbial community composition rather than with consistent changes in dominant taxa, overall bacterial load, or broad differences in alpha diversity. Notably, the detergent drawer emerged as the compartment most strongly associated with odour status. This compartment exhibited significantly higher Chao1 richness and Shannon diversity in odorous machines and showed the most consistent separation between odorous and non-odorous communities in beta-diversity analyses. These observations suggest that the detergent drawer may represent a key ecological niche for microbial processes linked to odour formation. However, the absence of pronounced differences in dominant taxa and total bacterial DNA concentrations indicates that odour development is likely driven by specific microbial populations and their metabolic activities rather than by overall community size or diversity alone. Furthermore, in our study, odour classification was based on user perception rather than standardized sensory evaluation or instrumental measurements. While this reflects real-life consumer experience, subjective odour assessment may not accurately represent actual odour intensity and therefore constitutes an important limitation of the present study. Future studies should therefore classify machines based on evaluations by professionally trained odour assessors and combine microbial community profiling with functional, metabolomic, and metatranscriptomic approaches. Finally, the relatively small number of sampled washing machines and the pronounced variability observed across several datasets limit the statistical power of the study, particularly for detecting small-to-moderate effects. Consequently, non-significant findings should not necessarily be interpreted as evidence for the absence of biological differences. Future studies including larger numbers of washing machines will be required to validate the observed trends and provide more robust estimates of effect sizes.

## Conclusion

This study provides a qualitative and quantitative assessment of bacterial communities in domestic washing machines categorized as odorous or non-odorous using full-length 16S rRNA nanopore sequencing and quantitative PCR. While microbial composition varied primarily by compartment, with detergent drawers showing the highest diversity and door seals dominated by *Moraxella osloensis*, differences between odorous and non-odorous machines were generally subtle. Notably, detergent drawer communities showed the strongest association with odour status across both alpha- and beta-diversity analyses, suggesting that this compartment may represent a key ecological niche associated with washing machine odour. These findings suggest that perceived odour is unlikely to be explained by major shifts in dominant bacterial taxa alone and may instead be influenced by microbial activity, subtle changes in community composition, or environmental factors. Future studies should incorporate larger machine cohorts, standardized odour evaluation by trained assessors, and integrative multi-omics approaches to better understand the biological and chemical mechanisms underlying laundry odour. Furthermore, future studies should collect user-related data, including detergent use, washing frequency, and household characteristics, to better assess environmental and behavioural factors potentially associated with odour formation, as also suggested by Jacksch et al. (2019).

## Data Availability

The raw sequencing data generated and analysed during the current study have been deposited in the NCBI Sequence Read Archive (SRA) under BioProject accession number PRJNA1480344. The data are currently under embargo and will be made publicly available upon publication of this article.
